# Site Specificity trial series (FR559) in *Pinus radiata* stands in New Zealand: Trial establishment, site description and initial data

**DOI:** 10.1016/j.dib.2026.112669

**Published:** 2026-03-10

**Authors:** Simeon J. Smaill, Amanda L. Matson, Loretta G. Garrett

**Affiliations:** a Ngāi Tahu Forestry, P.O. Box 13 046, Christchurch 8141, New Zealand; b Wageningen Environmental Research, Postbus 47, 6700 AA, Wageningen, the Netherlands; c Bioeconomy Science Institute, Private Bag 3020, Rotorua 3046, New Zealand

**Keywords:** Planted forests, *Pinus radiata*, Productivity, Tree nursery out planting

## Abstract

There is uncertainty over whether standardised bare-root tree nursery practices support optimal *Pinus radiata* seedling performance across the diverse site conditions of New Zealand’s planted forests. This article presents a dataset describing trial establishment, site characteristics, and initial soil conditions for the Site Specificity trial series (FR559), comprising 46 out-planting trials distributed across New Zealand’s planted forests. The trials were designed to evaluate how nursery production protocols interact with variation in site and soil properties to influence early seedling performance. Approximately 240,000 *P. radiata* seedlings were grown in a commercial nursery trial testing six fertiliser and six fungicide treatment combinations. Based on gains in growth metrics relative to standard practice, stock from three treatment combinations that outperformed standard practice were selected for post-nursery assessment alongside the standard practice. Seedlings from these treatments were planted into experimental plots at each of the 46 sites, which span a wide range of climatic, topographic, and edaphic conditions. The dataset includes site locations, trial design, and detailed soil physical and chemical properties measured at the time of establishment. These data provide a foundation for analysing interactions between nursery management, site conditions, and early tree health and performance. The breadth of sites represented enables assessment of site-specific responses, supports the development of experimental gradients in key environmental drivers, and provides a framework for future studies addressing site limitations to forest productivity. Overall, this dataset supports efforts to improve nursery production and establishment practices for enhancing early tree performance and resilience in New Zealand’s planted forests.

Specifications Table

**Table:** 

Subject	Earth & Environmental Sciences
Specific subject area	Sustainable productive planted forests through improved tree nursery management practices
Type of data	Tables and Figure
Data collection	The data were collected from field measurements and laboratory analysis of field collected soil samples. Plot slope was measured using a clinometer, aspect using a compass and altitude using a GPS. Soil samples collected at random points.
Data source location	Bioeconomy Science Institute, New Zealand. Location of 46 trials, latitude and longitudes, are in data tables.
Data accessibility	Data sets are provided with this article and available in the Figshare repository. Repository name: Figshare Data identification number: 10.6084/m9.figshare.30866348 Direct URL to data: https://doi.org/10.6084/m9.figshare.30866348.v1
Related research article	None

## 1 Value of the Data

• The data describes conditions across a series of 46 nursery out planting trials established across the New Zealand *P. radiata* estate.
• The data can be used to determine if the conditions at each trial site influences the early performance of *P. radiata* seedlings that were produced using different protocols in the nursery, and if any interactions between site conditions and nursery protocols are evident.
• The data will benefit New Zealand and global forestry science targeted towards a) the development of site-specific forestry practices, and b) continuously improving best management practices for the New Zealand and global forestry industry.
• The data can also be used to increase knowledge of the variation in planted forest soil properties in relation to early tree health and performance, providing a framework for additional trials and experiments addressing limitations associated with site specific factors.
• The wide range of sites represented by the data can also be used to support the establishment of experimental gradients in specific site factors to explore their impact on forest health and productivity.

## 2 Background

Management practices in any given New Zealand *Pinus radiata* bare-root tree nursery are largely standardised, treating stock the same way regardless of any variation in the conditions across the various locations that a nursery supplies. Given the known impact of nursery management on initial stock performance [1], there is value in exploring the potential for nursery management to produce stock that is pre-adapted to different environmental conditions. To address this, approximately 240,000 *P. radiata* tree seedlings (GF19) were grown in a commercial nursery using six combinations of fertiliser and six combinations of fungicide treatments. From these, three combinations were selected based on enhanced growth metrics relative to standard treatment, as this was considered critical for any uptake of changed practices. Stock from the selected treatment combinations (as well as the standard treatment) were then established into discrete plots at 46 sites around New Zealand. The stock was then grown on to determine if there was any variation in the correlation between site properties and stock performance (growth, survival) across the different nursery treatments.

The data provided in this article describes the location, climatic, topographic and soil properties of these 46 sites. This information is critical to determining which, if any, of the selected nursery protocols have enabled the *P. radiata* seedlings to be better or worse adapted to specific locations based on the properties of those sites.

## 3 Data Description

**Fig. 1:**
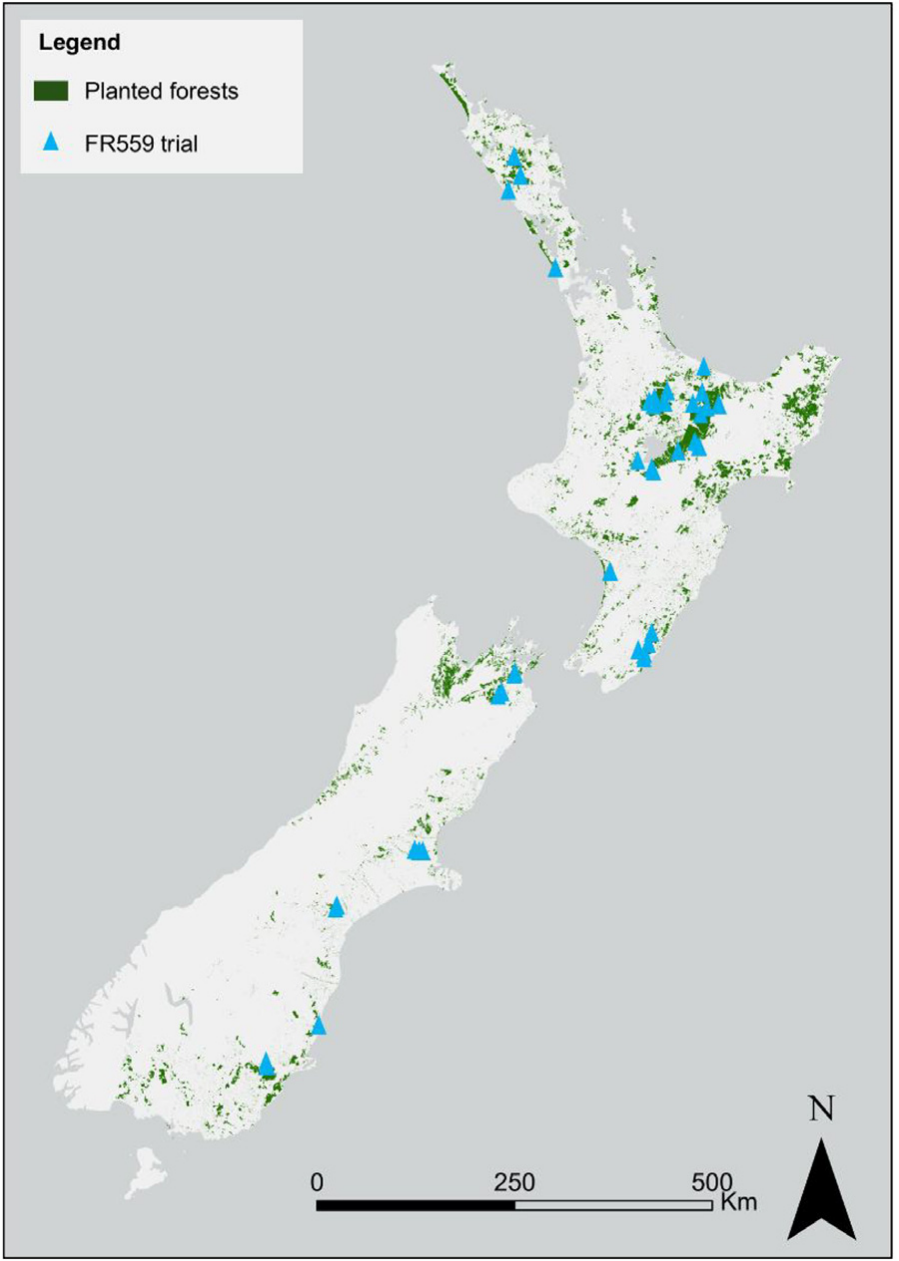
Location of the FR559 Site Specificity trials within New Zealand. Note some trials were located close together so cannot be resolved individually in the map.

The dataset contains raw and summarized data collected from 46 Site Specificity trials in planted *P. radiata* stands in New Zealand. Table 1 provides site characteristics for each trial, Table 2 lists the selected treatments applied to the stock at the *P. radiata* tree nursery, and Fig. 1 shows the location of the trials within New Zealand.

**Table 1:** Trial characteristics and trial series.

Trial ID	Latitude (°)	Longitude (°)	Mean air temp. (°C) [3]	Mean annual rainfall (mm) [3]	Elevation (m asl)	New Zealand Soil Classification [[4], [5], [6]]
FR559/01	-38.32	176.41	11.4	1312	439	Tephric Recent
FR559/02	-38.44	176.50	10.9	1255	545	Impeded Pumice
FR559/03	-38.38	176.59	11.4	1400	476	Orthic Pumice
FR559/04	-38.87	176.19	10.5	1665	642	Impeded Pumice
FR559/05	-38.76	176.45	10.4	1477	663	Impeded Pumice
FR559/06	-38.79	176.51	9.9	1462	774	Orthic Pumice
FR559/07	-38.31	176.77	12.9	1439	172	Orthic Pumice
FR559/08	-37.92	176.52	13.6	1617	180	Orthic Pumice
FR559/09	-38.19	176.51	12.2	2214	357	Tephric Recent
FR559/10	-38.19	176.03	11.3	1578	584	Orthic Podzol
FR559/11	-41.42	173.88	12.7	1194	47	Acid Brown
FR559/12	-41.43	173.88	11.7	1082	362	Acid Brown
FR559/13	-41.63	173.69	11.9	648	223	Immature Pallic
FR559/14	-41.67	173.66	11.3	823	275	Rocky Recent
FR559/15	-43.46	172.42	11.5	652	74	Fluvial Recent
FR559/16	-43.47	172.49	11.6	614	48	Fluvial Recent
FR559/17	-43.45	172.36	11.5	698	103	Firm Brown
FR559/18	-44.12	171.08	10.3	753	198	Perch-gley Pallic
FR559/19	-44.08	171.10	9.3	817	542	Orthic Brown
FR559/20	-45.45	170.80	10.4	589	54	Argillic Pallic
FR559/21	-45.48	170.80	10.5	581	59	Gley Raw
FR559/22	-45.85	169.94	8.6	880	436	Orthic Brown
FR559/23	-45.86	169.93	8.4	883	442	Orthic Brown
FR559/24	-45.90	169.95	8.3	901	443	Orthic Brown
FR559/25	-45.91	169.95	8.4	902	442	Orthic Brown
FR559/26	-35.50	173.83	14.9	1711	183	Orthic Granular
FR559/27	-35.61	173.93	14.6	1592	133	Oxidic Granular
FR559/28	-35.72	173.90	14.9	1431	96	Oxidic Brown
FR559/29	-35.89	173.76	15.0	1200	66	Pan Podzol
FR559/30	-38.28	175.84	12.1	1519	392	Orthic Pumice
FR559/31	-38.32	175.85	12.4	1527	334	Orthic Pumice
FR559/32	-38.34	175.83	12.5	1531	251	Impeded Pumice
FR559/33	-38.31	176.00	12.2	1525	360	Orthic Pumice
FR559/34	-40.19	175.34	13.3	912	49	Sandy Brown
FR559/35	-41.19	175.85	12.2	1534	288	Orthic Brown
FR559/36	-41.04	175.90	12.1	1171	260	Orthic Brown
FR559/37	-41.16	175.82	12.1	1522	268	Orthic Brown
FR559/38	-36.79	174.43	14.2	1328	39	Sandy Recent
FR559/39	-36.79	174.43	14.2	1328	38	Sandy Recent
FR559/40	-36.79	174.43	14.2	1328	36	Sandy Recent
FR559/41	-39.09	175.83	10.7	2038	542	Orthic Pumice
FR559/42	-39.09	175.83	10.7	2038	545	Orthic Pumice
FR559/43	-38.99	175.62	10.3	2172	597	Orthic Pumice
FR559/44	-40.93	175.93	12.5	1026	146	Orthic Brown
FR559/45	-40.92	175.94	12.8	1023	141	Orthic Brown
FR559/46	-41.13	175.81	12.3	1321	236	Argillic Pallic

**Table 2:** Tree nursery treatments. Full descriptions of the treatments are given in reference [2].

Treatment	Pre-seed drilling fertiliser	Fungicide application	Post-seed drilling fertiliser	Seedling root collar diameter (mm)
A	Standard	Standard	Standard	6.52 ± 0.16
B	Standard	Standard	50% nitrogen rate applied using biuret, otherwise standard	6.93 ± 0.19
C	Agroblen	Reduced	Standard	7.01 ± 0.11
D	Bioboost	Reduced	Standard	6.88 ± 0.11

The raw data on initial soil and trial details for the Site Specificity trial series (FR559) in *P. radiata* stands in New Zealand are stored in Figshare [2]: https://figshare.com/s/1067e14ab95952548047.

## 4 Experimental Design, Materials and Methods

### 4.1 Site selection

Candidate locations for trial establishment were identified in partnership with multiple commercial forestry companies, regional councils and various landowners. Key criteria for site selection were.

• At the broad scale, each location contributed sufficient variation in properties to generate a national-level climatic and altitudinal gradient within areas of production forests.
• At the local scale, there was potential to establish small clusters of trial locations in a given area that would represent substantial variation in soil and topographical properties while also allowing logistical efficiency.

This process identified a pool of 54 trial locations suitable for the establishment of a trial. This was refined to 46 following further filtering based on logistical and resourcing considerations. The FR559 trial series was planted at the 46 locations over 2017-2018.

### 4.2 Trial design

All trials were comprised of 12 sub-plots, representing three replicates of each of the four selected nursery treatments using GF19 (Growth and Form) seedlings (Table 2). The sub-plots at a given trial were laid out to hold either 15 seedlings (all sub-plots on a 3×5 layout) or 16 seedlings (all sub-plots on a 4×4 layout), the key driver of this was the stocking rate used to establish the trial which varied from 833 trees per ha to 1325 trees per hectare, based on the preference of the land owner. This made a total of either 180 or 192 trees per trial.

The sub-plots for each trial were located to ensure uniformity across the local topography; this was generally achievable with a contiguous 3×4 or 6×2 grid, but in some cases variation in slope or other landform property required the sub-plot layout to be split over a gully or other feature to produce consistent characteristics. Pegs were installed to mark the corner of each plot to support accurate planting and subsequent soil sampling and tree measurements. Prior to planting, sub-plots were blocked into three groups of four sub-plots, with each group comprised of one of each of the four nursery treatments that was randomly assigned to a sub-plot. The trial was then planted out with the nursery stock according to the installed design.

### 4.3 Time zero soil sampling, preparation and testing

Mineral soil samples were collected from each trial at the time of tree planting. Soil samples were collected using a stainless steel 25 mm diameter Hoffer tube sampler at depth increments of 0-10, and 0-30 cm depth next to the planted seedling. One sample was collected from each of the 12 sub-plots and bulked by depth increment to make a composite sample representative of the trial area. Bulk density samples were collected next to a selected planted tree (on the tree spot or rip mound if present) from sub-plot 1 (sub-plot 3 for FR559/13) at depth increments of 0-10, 10-20, 20-30 cm and from sub-plot 6 and 9 for 0-10 cm depth increment. Soil bulk density was collected from 33 of the 46 trials. Where there was site preparation prior to planting (e.g., spot mounding or rip mounding) the soil samples were collected from the top of the mound near the planted tree. Where there was no site preparation the soil samples were collected near the planted seedling.

Soil samples were air-dried (<40°C) then sieved to retain the <2 mm and greater >2 mm mineral soil fraction and >2 mm organic material fraction. Subsamples of the <2 mm and >2 mm mineral fractions were archived. The soil bulk density fractions were oven dried at 104°C and weighed. The 2 mm fraction of the soil samples were analysed for total carbon and total nitrogen using a LECO FPS-21,000 CNS thermal combustion furnace; pH measured by 1:2.5 soil/water suspension; available phosphorus using FIA colorimetry after sequential 1:10 Bray 2 (NH4F/HCl) extraction; available elements (aluminium, boron, calcium, copper, iron, magnesium, manganese, sodium, phosphorus, potassium, zinc) using ICP-MS (inductively coupled plasma mass spectrometry) after Mehlich 3 extraction; total phosphorus using FIA colorimetry after sulphuric acid digest. All reported on an oven-dry (104°C) basis. Sulphate-S was measured by IC (Ion Chromatography) after 0.02 M potassium phosphate extraction and reported on an air-dry basis of 35–40°C. Elemental totals aluminium, arsenic, boron, barium, calcium, cadmium, cobalt, chromium, copper, iron, potassium, magnesium, manganese, sodium, nickel, phosphorus, lead, sulphur, selenium, strontium, thallium, uranium, vanadium, and zinc (Al, As, B, Ba, Ca, Cd, Co, Cr, Cu, Fe, K, Mg, Mn, Na, Ni, P, Pb, S, Se, Sr, Tl, U, V, and Zn) as measured by ICP-MS (inductively coupled plasma mass spectrometry) using a modified USEPA digestion method 3050B to include a reverse aqua regia digest of 1 HCl: 3 HNO_3_ to limit Cl− interference and reported on an oven dry basis of 50°C.

Diffuse reflectance MIR spectra were acquired for all <2 mm chemistry samples using methods described in Garrett et al. [7]. In summary, a representative 10 g sub-sample of soil (<2 mm fraction) was ground for 180 s in a 45 ml zirconia ceramic grinding vial containing two 12.7 mm zirconia ceramic balls using a Spex800D mixer mill. All samples were run in replicates of four for diffuse reflectance MIR spectra using a Bruker Invenio-S Fourier transform infrared (FTIR) spectrometer with a Bruker HTS-XT (High Throughput Screening Extension) microplate reader fitted with a liquid nitrogen cooled mercury cadmium telluride detector. The system was purged with CO_2_-free dry air using a Peak Scientific PG14L generator. Sample spectra were acquired in diffuse reflectance mode with 50 co-added scans from 8000 to 400 cm^−1^ at 4 cm^−1^ resolution. The spectra were pre-processed using Bruker OPUS 8.2 software applying the make scalar compatible and cut functions to truncate the wavenumber range to 4000 to 600 cm^−1^.

## Limitations

The trial locations were biased towards existing planted *P. radiata* productive forestry land use, with limited number of sites representing first rotation planted forests (previous land-use productive pasture/grazing). This reduces the applicability of the data to studies comparing land-use change.

Soil sampling was biased towards areas within the trials directly adjacent to tree planting, not between the rows of trees. This provided data on the conditions in the immediate vicinity of the young trees, but it is acknowledged that randomized sampling locations may be more suitable for characterizing the entire trial area.

## Ethics Statement

The authors have read and follow the ethical requirements for publication in Data in Brief and confirming that the current work does not involve human subjects, animal experiments, or any data collected from social media platforms.

## CRediT Author Statement

**Simeon J Smaill:** Conceptualization, Methodology, Investigation, Data curation, Writing –original draft, Writing –review & editing, Supervision; **Amanda L Matson:** Methodology, Investigation, Data curation, Writing –review & editing; **Loretta G Garrett:** Methodology, Investigation, Data curation, Writing –original draft, Writing –review & editing.

## Data Availability

FigshareRaw data on initial soil and trial details for the Site Specificity trial series (FR559) in Pinus radiata stands in New Zealand (Original data).
